# Anti-arthritic activities of cross-linked hyaluronic acid-dexamethasone hydrogel in a rat model of gouty arthritis

**DOI:** 10.3389/fphar.2026.1810048

**Published:** 2026-05-13

**Authors:** Jingya Zhao, Peichu Tian, Binsheng Li, Yuqi Yang, Mengyu Fu, Tao Zhang, Zhiwei Zhang, Li Wu

**Affiliations:** 1 Department of Endocrinology, Taiyuan People’s Hospital, Taiyuan, China; 2 Shanxi Key Laboratory of Bone and Soft Tissue Injury Repair, Department of Orthopedics, The Second Hospital of Shanxi Medical University, Taiyuan, China; 3 College of Basic Medicine, Shanxi Medical University, Taiyuan, China

**Keywords:** cartilage, cross-linked hyaluronic acid hydrogel, dexamethasone, gouty arthritis, inflammatory factors, lipopolysaccharide, monosodium urate, synovitis

## Abstract

**Background:**

Gouty arthritis (GA) is a type of inflammatory joint disease in which the deposition of monosodium urate (MSU) crystals inside the joints leads to severe inflammatory reactions, such as synovitis. Cross-linked hyaluronic acid hydrogel (cHA gel) has been shown to have a chondroprotective effect, while dexamethasone (Dex) is an anti-inflammatory drug; both have been used in clinical practice to a certain extent. This study was designed to compare the therapeutic effects of cHA gel pre-mixed with Dex (cHA-Dex gel) or without Dex (cHA gel alone) in a rat model of GA.

**Methods:**

In this study, 2-month-old male Sprague-Dawley (SD) rats were used. The GA model was established by intra-articular injection of a solution containing 40 mg/mL MSU crystals and 0.1 mg/mL lipopolysaccharide (LPS) dissolved in saline, and this procedure was repeated every 2 weeks. The rats were subsequently randomly allocated into four groups (*n* = 8 per group): the MSU/LPS + saline group, the MSU/LPS + cHA gel group, the MSU/LPS + cHA gel containing 0.5 mg/mL Dex (cHA-Dex gel) group, and a negative control group. One day after MSU/LPS injection, the designated treatments were administered via intra-articular injection only once. Various examinations were performed 1 day after the fifth MSU/LPS induction. Behavioral indicators included the degree of knee joint swelling, the capacity of the limb to bear weight, grip strength, mechanical pain threshold, acetone test results, and the gait scoring. Imaging and histological assessments included micro X-ray, Indian ink staining, safranin O-fast green staining, immunohistochemistry, and synovial hematoxylin-eosin (HE) staining. Biochemical and molecular biological markers were evaluated by serum enzyme-linked immunosorbent assay (ELISA) and quantitative real-time polymerase chain reaction (RT-qPCR). The detected targets included interleukin-1β (IL-1β), interleukin-6 (IL-6), interleukin-10 (IL-10), tumor necrosis factor α (TNF-α), matrix metalloproteinase-3 (MMP-3), and matrix metalloproteinase-9 (MMP-9) expression levels. Eight rats per group (n = 8) were used for all assays, with cartilage from two rats pooled to yield four biological replicates per group (n = 4) for RT-qPCR.

**Results:**

The findings showed that the deposition of intra-articular MSU/LPS led to pathological changes, including inflammation and cartilage damage. Micro X-ray scans showed that the knee joint structure treated with cHA-Dex gel had a much better level of integrity than the MSU/LPS + saline and MSU/LPS + cHA gel groups. In the MSU/LPS + cHA-Dex gel group, Indian ink staining was less, and safranin O-fast green staining showed an increase in proteoglycan (PG) content and a decrease in the extent of cartilage damage. Histological quantitative analysis showed statistically significant differences in the relevant scores (*p* < 0.05). Immunohistochemical staining indicated strong positive staining for type II collagen (COL II), while expression levels of MMP-3 and MMP-9 were downregulated. HE staining of synovial tissue showed that the synovial lining cell layer became thinner, with fewer infiltrating inflammatory cells. Analysis of serum and cartilage tissues showed that cHA-Dex gel significantly lowered the level of inflammatory factors.

**Conclusion:**

Altogether, the cHA gel combined with Dex demonstrated superior therapeutic outcomes in comparison to cHA gel alone in a rat model of GA. The presented work provides preclinical evidence for a localized cHA-Dex gel delivery strategy that may complement existing clinical treatments for GA, with further validation required for translational application.

## 1 Introduction

Gouty arthritis (GA), a chronic inflammatory joint disorder, is becoming more common with aging. Pain and reduced function are its main symptoms (Ibrahim Fouad et al., 2025). GA pathology is characterized by synovial inflammation, cartilage degradation and bone spur formation; therefore, the damage sites include synovium, cartilage, and subchondral bone (Chen et al., 1999; Yin et al., 2014), particularly in the setting of recurrent gout attacks in the knee joint (Accart et al., 2022). A malfunction in purine metabolism combined with impaired uric acid excretion results in the accumulation of monosodium urate (MSU) crystals in the joints and their surrounding tissues. These needle-shaped MSU crystals can trigger an innate immune response that results in the release of inflammatory mediators (Li et al., 2021). This cascade leads to synovitis, cartilage degradation, bone erosion, and cartilage calcification (Terkeltaub et al., 2009). During recurrent GA attacks, patients often experience redness, swelling, severe pain, and edema; hence quick symptom relief is the goal of the therapy. Subsequently, the traditional medications, such as nonsteroidal anti-inflammatory drugs, colchicine, and glucocorticoids, effectively manage the symptoms of GA. Due to drug limitations, such as gastrointestinal adverse reactions, cardiovascular risks as well as drug resistance, which further restrict their long-term use, short-term and low-dose usage is recommended (Guo et al., 2022). Thus, there is a need for safer, longer-acting, lower hepatorenal toxicity GA intervention strategies.

Evidence suggests that low molecular weight hyaluronic acid (LMW-HA) possesses anti-inflammatory, antioxidant, and uric acid-lowering effects *in vivo*, indicating its potential for treating GA and hyperuricemia (Li et al., 2019). Intra-articular injection of high molecular weight hyaluronic acid (HMW-HA) can alleviate pain and pain-related behaviors in rats with GA (Marcotti et al., 2018). Beyond its inherent therapeutic properties, HMW-HA can also serve as a drug delivery vehicle for the treatment of GA (Liu et al., 2022; Sathiyamoorthy et al., 2025). The chain length of LMW-HA generally lies between 0.5 and 3.6 million Da, whereas that of chemically cross-linked high molecular weight hyaluronic acid (cHA) can be elevated to 6.0 million Da (Zhang et al., 2016). The native LMW-HA can only last about 10–13 h in the joint cavity; however, the retention time of cross-linked formulations may be prolonged to 8.8 ± 0.9 days (Larsen et al., 2012). The quick metabolism and excretion of LMW-HA result in the limitation of clinical benefits in certain patients. A recently developed cHA gel exhibits a longer retention time than LMW-HA gel, stimulates the generation of more endogenous HA, provides superior pain relief, has a better cost-benefit ratio, and causes fewer related adverse reactions (Zhang et al., 2024).

Intra-articular glucocorticoid injection has been a longstanding clinical practice. Dexamethasone (Dex) is considered one of the most effective agents among contemporary glucocorticoids (Madamsetty et al., 2022). These drugs act by blocking the matrix degradation caused by inflammatory mediators. Consequently, great protective effects on cartilage under inflammatory conditions have been obtained from various experimental and clinical evidence (Wang et al., 2021; Di Francesco et al., 2022). In our previous works (Zhang et al., 2016; Zhang et al., 2024), cHA-Dex hydrogel (cHA-Dex gel) was prepared through the physical mixing of cHA gel and Dex. This dosage form was used for therapeutic intervention of post-traumatic osteoarthritis (PTOA). In our previous studies of PTOA, sustained release of Dex from cHA-Dex gel was shown to reduce cartilage inflammation, reverse Dex-enhanced chondrocyte apoptosis, and slow PTOA progression. Nevertheless, how effective it is against GA has not been investigated yet. Therefore, our study aimed to investigate the anti-inflammatory effects of the cHA-Dex gel in GA rats.

## 2 Materials and methods

### 2.1 Reagents

Uric acid and lipopolysaccharide (LPS) were purchased from Sigma-Aldrich (St. Louis, MO, USA). MSU crystals were prepared as previously described (Marcotti et al., 2018). cHA gel was obtained from BioRegen Biomedical Company (Changzhou, China). Dex was bought from Shuanghe Pharmaceutical Company (Wuhan, China). Antibodies against COL II (type II collagen), MMP-3 (matrix metalloproteinase 3), and MMP-9 (matrix metalloproteinase 9) were purchased from Cell Signaling Technology (Danvers, MA, USA). Enzyme-linked immunosorbent assay (ELISA) kits for interleukin-1β (IL-1β), interleukin-6 (IL-6), interleukin-10 (IL-10), tumor necrosis factor α (TNF-α), matrix metalloproteinase-3 (MMP-3), and matrix metalloproteinase-9 (MMP-9) were obtained from SenBeiJia Biological Technology Co., Ltd. (Nanjing, Jiangsu, China). The TRIzol reagent, iScript™ cDNA synthesis kit, and QuantiTect SYBR Green PCR kit were bought from Invitrogen (Carlsbad, CA, USA).

### 2.2 Animals

Male Sprague-Dawley (SD) rats (clean grade), aged 2 months and weighing 180–220 g, were employed in the present study. The rats were kept in the Laboratory Animal Center of Shanxi Medical University under controlled environmental conditions: the temperature was 20 °C, the humidity was 45%, and the lighting was a 12-h light/12-h dark cycle, with continuous availability of food and water. All animal experimental procedures were approved by the Animal Care and Use Committee of Shanxi Medical University (Approval No.: 2021-102), and all experiments were conducted in strict accordance with the ARRIVE guidelines.

### 2.3 Induction of gouty arthritis and experimental grouping in a rat knee joint model

**FIGURE 1:**
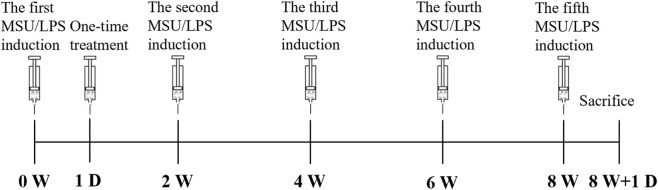
Timeline of induction of repeated gouty arthritis, intervention administration and subsequent evaluation time points.

Thirty-two SD rats were randomly assigned to four groups (8 rats per group): (1) MSU/LPS + Saline, (2) MSU/LPS + cHA gel, (3) MSU/LPS + cHA-Dex gel (0.5 mg/mL Dex), and (4) Negative Control (no MSU/LPS induction). The cHA gel and cHA-Dex gel were pre-mixed, and then sterilized by autoclaving. Following anesthesia and routine disinfection, the right knee joint of rats in groups 1–3 was injected with 50 µL of a solution containing 40 mg/mL MSU crystals and 0.1 mg/mL LPS dissolved in saline using a 26-gauge needle, and this procedure was repeated every 2 weeks (Accart et al., 2022). LPS is used to recapitulate the secondary inflammatory amplification observed in human GA flares (often driven by sterile inflammation and innate immune activation, not bacterial endotoxin). The 0.5 mg/mL Dex concentration was selected as a starting point based on our prior PTOA work (where it demonstrated chondroprotection without overt cytotoxicity) and that this dose was chosen to avoid excessive Dex exposure in the initial GA model characterization (Zhang et al., 2016; Zhang et al., 2024). After each injection, the rat was kept under observation to confirm that no suspension had leaked out and that it was all retained uniformly in the joint cavity (Marcotti et al., 2018). One day after the first MSU/LPS injection, rats in groups 2 and 3 received only one injection of 50 µL cHA gel or cHA-Dex gel, respectively. In order to rule out any effect caused by the injection procedure alone, rats in Groups 1 and 4 were given an injection of 50 µL normal saline into the right knee joint at the same time points as the groups 2 and 3. As shown in Figure 1, all rats were euthanized 1 day after the fifth MSU/LPS induction, according to previously reported histological changes in the knee joints of GA rats (Accart et al., 2022). The tibial plateau was taken for histological examination, and the femoral condyle was taken for mRNA expression analysis.

### 2.4 Measurement of knee joint diameter

The mediolateral width of the knee joint was determined using a digital display micrometer. Joint width was measured 1 day after the fifth MSU/LPS induction.

### 2.5 Behavioral testing

All behavioral tests commenced after the rats were acclimatized to the testing room for at least 3 h and to the specific apparatus for an additional 1 h. All behavioral testing was performed by one experimenter with no knowledge of the treatment groups. Animal grouping was randomized using a computer-generated sequence. The behavioral evaluation included the following metrics: weight-bearing asymmetry, grip strength, mechanical pain threshold, and the acetone test. The weight-bearing capacity of animals was tracked over time by an incapacitation tester (Bioseb, Vitrolles, France): the rats were placed in an inclined chamber in such a way that each hind paw was resting on a separate force-sensitive platform. The load borne by one limb (g) was determined within 5 s, and the procedure was performed 6 times to determine the average. The results were given as an asymmetry index determined from the formula: index = (weight-bearing of the unaffected limb–weight-bearing of the affected limb)/total weight of both limbs ✕100%. An increase in this index indicates mechanical hyperalgesia. Grip strength was evaluated with the use of an Insight grip strength meter (Ribeiro Preto, Brazil), and the highest pulling force (g) was recorded, as previously described (Hoss et al., 2023).

The mechanical pain threshold was quantified by means of a digital von Frey aesthesiometer (Insight, Ribeiro Preto, Brazil), with force expressed in grams (g). Rats were confined in transparent cylinders set over a metal grid. The plantar surfaces were stimulated with the filament vertically for 1 s. Rapid withdrawal of the tested hind paw was regarded as a positive reaction. The two hind paws were tested one after another, and the force at which a 50% withdrawal probability was observed was considered to be the pain threshold. The results were expressed as the difference between the post-treatment threshold and the baseline. A decrease in threshold (negative value) indicated the development of mechanical hyperalgesia.

To assess cold stimulus sensitivity, the acetone test was employed (Marcotti et al., 2018): the rats were set on a metal mesh platform inside circular plastic chambers, and 100 µL of acetone was dropped in series onto the plantar surfaces of both hind paws. The frequency of paw lifting, licking, biting, shaking, or guarding behaviors was scored over 60 min. The data were presented as the ratio of post-treatment to pre-treatment values; a ratio of about 1 meant no cold hyperalgesia, whereas a higher ratio meant increased cold pain sensitivity. Both measures were taken in triplicate twice (total 6 repetitions), and the mean value was used for further work.

### 2.6 Gait score assessment

The observation and evaluation of hindlimb functional deficits were done by a double-blinded gait scoring system, which was consistent with the standards reported previously (Ibrahim Fouad et al., 2025). In this investigation, a 4-grade scoring system was created as follows: Grade 1 - the gait pattern does not show any remarkable difference from the normal state; Grade 2 - the hindlimbs have mild instability during the movement, or the distance between the two feet is increased compared with the normal condition; Grade 3 - marked ataxia is present, with foot abduction and limb abduction during walking; Grade 4 - there is severe dysfunction of the hindlimbs, which can no longer be normally used for bearing weight, and foot abduction is very evident.

### 2.7 Radiography

Bilateral knee joint radiographs were taken using a Faxitron Bioptics micro-X-ray system (Lincolnshire, Illinois, USA) just before the rats were euthanized. Periarticular calcifications of the rat knee joints were graded by a radiographic grading system published previously (Innes et al., 2004). Grade 0 is given when no abnormalities are found; Grade 1 corresponds to slight calcific lesions, Grade 2 to moderately calcific lesions, and Grade 3 to severely calcific lesions. Three independent observers, who were trained by clinicians with extensive clinical experience, conducted all scoring procedures. To keep the assessors’ judgments completely unbiased, the observers were kept unaware of which group each rat belonged to before scoring. We used the mean score from three independent observers as the final score for each sample.

### 2.8 Histological assessment

Eight rats per group had their tibial plateaus stained with Indian ink after their knee joints were harvested. Under a stereomicroscope, the extent of cartilage fibrosis and defects was recorded using the Meachim grading system (Meachim, 1972). Indian ink-stained specimens were fixed for 3 days in 10% neutral formalin, then decalcified for 5 days in a 20% ethylenediaminetetraacetic acid solution before being embedded in paraffin. Following standard procedures, continuous sections were cut along the tibial plateau’s sagittal axis for double staining with safranin O and fast green. Histological lesions were assessed using the modified Osteoarthritis Research Society International (OARSI) scoring scale (Gerwin et al., 2010). Three independent observers, blinded to the treatment groups, scored the sections. Mean scores from three independent observers were taken as the final score for each sample.

### 2.9 Immunohistochemical assay

Immunostaining of paraffin sections was done using the Histostain-SP kit (Invitrogen, Carlsbad, California, USA) to detect the protein expression of COL II, MMP-3, and MMP-9. Firstly, sections were incubated with 5 mg/mL hyaluronidase (Sigma-Aldrich, St. Louis, MO, USA) for 20 min at 37 °C to unmask antigens. Subsequently, potential sites of non-specific binding were blocked using LICOR blocking serum. The sections were exposed to primary antibodies overnight at 4 °C (Santa Cruz, CA, USA). Sequential addition of biotinylated secondary antibody and streptavidin-peroxidase complex was performed the following day, and color development was conducted with 3′3-diaminobenzidine. Morphological photographs were taken using a bright-field microscope (Nikon E800, Melville, NY, USA) equipped with a digital camera.

### 2.10 Histopathological assessment of synovitis

Staining of sagittal joint sections was performed with routine hematoxylin-eosin staining (HE) to evaluate the degree of synovial inflammation. Semi-quantitative evaluations of synovial lining thickness, subsynovial stroma proliferation, and the number of infiltrating inflammatory cells were done in accordance with the OARSI synovitis histological criteria (Gerwin et al., 2010). Three independent observers, who were blinded to the treatment groups, scored the sections. The mean of their scores was used as the final score for each sample.

### 2.11 Assessment of serum inflammatory cytokine concentrations

One day after the fifth MSU/LPS induction, serum samples were collected from the abdominal aorta of rats, and the blood was then centrifuged at 3500 r/min for 15 min to separate serum. The levels of these proteins (IL-1β, IL-6, IL-10, TNF-α, MMP-3, and MMP-9) were measured strictly following the operating instructions of the ELISA kit.

### 2.12 Quantitative real-time polymerase chain reaction (RT-qPCR) detection

Femoral condylar cartilage was taken from all rats and then ground into powder. Cartilage samples were carefully dissected with a scalpel from two separate rats and then pooled to form a single sample; thus, each group contained four pooled samples for RT-qPCR analysis, corresponding to a total of 8 rats per group (n = 8/group). The total RNA was reverse transcribed into complimentary DNA (cDNA) using a commercial reverse transcription kit. PCR amplification was performed using the synthesized cDNA as template with a commercial PCR amplification kit. 18S rRNA was selected as an internal control based on its stability in rat cartilage under inflammatory conditions, as previously validated in our laboratory (unpublished data) and its high abundance in low-yield cartilage RNA samples (a critical practical consideration for this study). Expression levels of 18S were confirmed to be stable across treatment groups in preliminary experiments (Ct variation <0.5 cycles). The Ct values of the target genes were determined by computer software. Each experimental condition was repeated 6 times. Primer sequences are available in Supplementary Table S1.

### 2.13 Statistical analysis

Sample size was determined based on a prior power analysis using G*Power software (version 3.1.9.2). According to Cohen’s criteria, an effect size of 0.8 is considered “large” and is appropriate for proof-of-concept studies where a robust therapeutic effect is expected. Assuming an effect size of 0.8, α = 0.05, and power = 0.80, a minimum of seven animals per group were required. To account for potential attrition, eight rats per group were included.

SPSS 20.0 was used to carry out the statistical analyses. All data are expressed as the mean ± standard deviation (SD). Data for radiographic grading, Meachim grading, OARSI cartilage scores, and OARSI synovitis scores were analyzed using the Kruskal–Wallis test followed by Dunn’s *post hoc* test. Differences among multiple groups of continuous data were determined by one-way analysis of variance (ANOVA) followed by Tukey’s tests. A *p*-value less than 0.05 was regarded as statistically significant.

## 3 Results

### 3.1 Effect of cHA-Dex gel on knee joint swelling in GA rats

**FIGURE 2:**
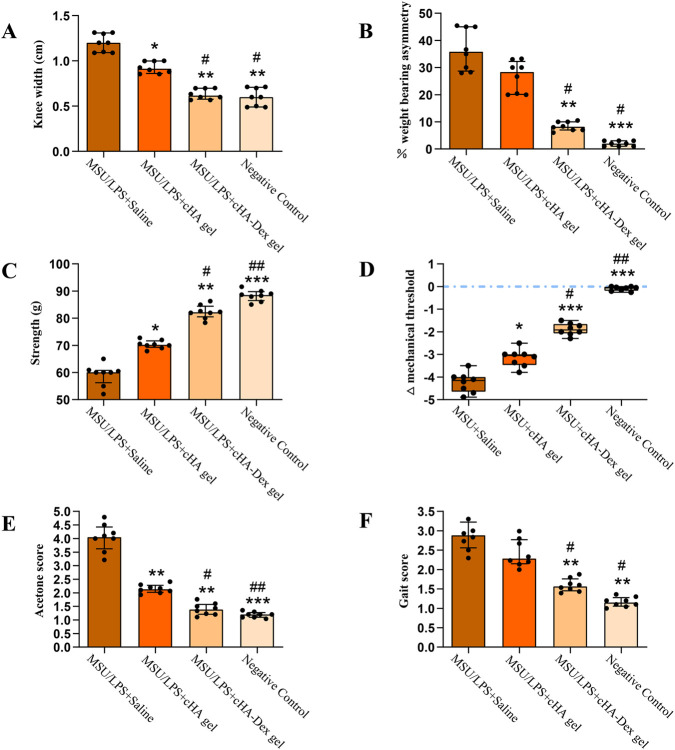
Effects of interventions in different experimental groups on monosodium urate (MSU)/lipopolysaccharide (LPS)-induced knee joint swelling **(A)**, weight-bearing capacity **(B)**, grip strength **(C)**, mechanical pain threshold **(D)**, cold stimulus sensitivity **(E)**, and gait score **(F)** in rats. Data are presented as mean ± SD. *p < 0.05, **p < 0.01, and ***p < 0.001 compared to the MSU/LPS + Saline group; #*p* < 0.05, and ##*p* < 0.01 compared to the MSU/LPS + cHA gel group; n = 8.

Assessment of knee joint swelling was carried out 1 day after the fifth intra-articular injection of MSU/LPS. Rats treated with the MSU/LPS suspension experienced a significant increase in knee joint width compared to the control group, thus, the presence of joint swelling was confirmed (Figure 2A). In order to assess the effectiveness of the therapeutic interventions, the knee joint width of GA rats in the treatment groups was also measured at the same time points. It was found that the MSU/LPS + cHA gel group and the MSU/LPS + cHA-Dex gel group were able to diminish the extent of knee joint swelling in rats, and the alleviation of symptoms in the MSU/LPS + cHA-Dex gel group was significant as compared to the MSU/LPS + cHA gel group (*p* = 0.02). The results presented here strongly support the notion that cHA gel and cHA-Dex gel treatment can alleviate joint edema and inflammatory responses induced by MSU/LPS. Moreover, in a rat model of GA, cHA-Dex gel was proven to be more effective than cHA gel in alleviating MSU/LPS-induced joint edema and swelling.

### 3.2 Effects of cHA-Dex gel on weight-bearing, grip strength, mechanical pain threshold, acetone test responses, and gait scoring in GA rats

Prior to MSU/LPS injection, hind limb weight distribution was practically the same between the two hind limbs of rats. However, following MSU/LPS administrations, there was a statistically significant increase in the asymmetry of weight-bearing between the hind limbs (to about 36%) in the MSU/LPS + Saline group, such that the non-injected hind limb was supporting approximately 64% of the total body weight. Treatment with cHA-Dex gel significantly restored weight-bearing symmetry (Figure 2B); statistical analysis revealed that this effect was different from both the MSU/LPS + Saline group and the MSU/LPS + cHA gel group. Grip force data (Figure 2C) showed that when compared to the MSU/LPS + Saline group, grip force was significantly higher in the negative control group and each treatment group, with statistical significance being reached in all comparisons. Furthermore, grip force in the MSU/LPS + cHA-Dex gel group was significantly greater than that in the MSU/LPS + cHA gel group (*p* = 0.02). The mechanical nociceptive threshold of the injected hind limb paw, assessed by von Frey testing, was markedly lower in rats that had received intra-articular MSU/LPS (Figure 2D). On the other hand, the negative control group and all the treatment groups had significantly higher mechanical nociceptive thresholds compared to the MSU/LPS + Saline group, with statistically significant differences observed in each. Furthermore, the MSU/LPS + cHA-Dex gel group exhibited a significantly higher mechanical nociceptive threshold compared to the MSU/LPS + cHA gel group (*p* = 0.03). The absolute baseline values of mechanical nociceptive threshold were as follows: MSU/LPS + Saline group 15.42 ± 1.61 g, MSU/LPS + cHA gel 14.84 ± 1.42 g, MSU/LPS + cHA-Dex gel group 15.10 ± 1.06 g, the negative control group 15.65 ± 1.31 g, with no significant between-group differences (*p* = 0.87). Cold hyperalgesia induced by acetone application was observed in the paws of the injected hind limbs. Consistent with the patterns observed for mechanical nociceptive thresholds, the negative control group and all treatment groups showed better responses than the MSU/LPS + Saline group (Figure 2E).

Gait quality was thoroughly assessed using a gait scoring system. Rats in the negative control group maintained a normal gait throughout the experiment without any signs of abnormality. Rats with GA induced by MSU/LPS showed severe gait abnormalities and walking difficulties associated with typical signs such as swelling and lameness of the knee joint. Statistical analysis revealed that the gait score of rats in the MSU/LPS-induced group was markedly elevated compared to that in the negative control group, being almost twice as high as the latter (Figure 2F). The gait scores of MSU/LPS-induced GA rats treated with cHA-Dex gel exhibited a significantly lower level than those of both the MSU/LPS-only group and the cHA gel-treated group. These data indicate that cHA-Dex gel treatment is more effective than cHA gel in therapeutically improving MSU/LPS-induced gait disorder in GA rats.

### 3.3 Effect of cHA-Dex gel on radiographic change in the knee of GA rats

**FIGURE 3:**
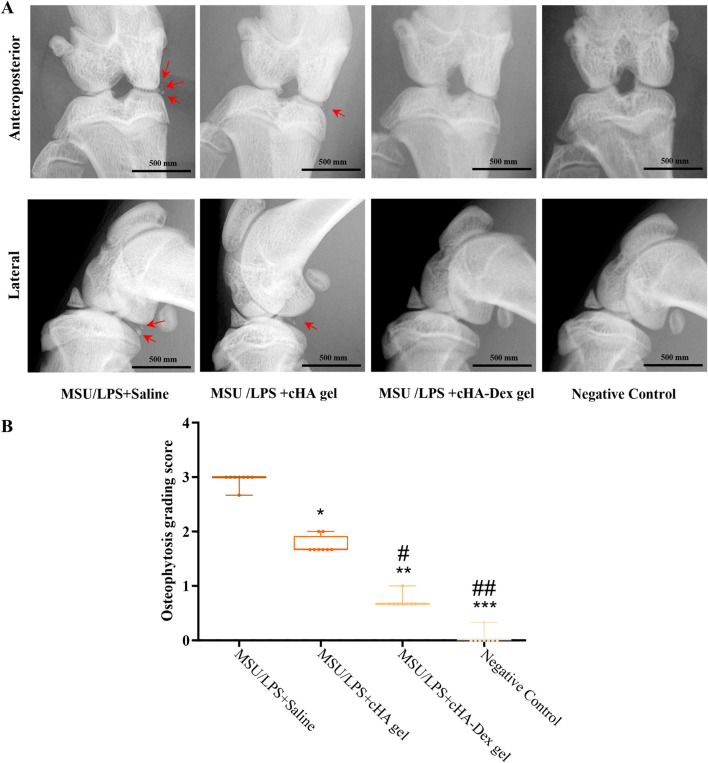
Micro X-ray imaging **(A)** was used to assess degenerative changes in the right knee joint. **(B)** Radiographic classification was performed using a subjective grading scale from 0 to 3 points, based on the extent of calcified foci in the knee joint. Data are presented as mean ± SD. *p < 0.05 and ***p < 0.001 compared to the MSU/LPS + Saline group; #*p* < 0.05 and ##*p* < 0.01 compared to the MSU/LPS + cHA gel group; n = 8.

Micro X-ray was performed on GA rat knee to assess the existence and degree of imaging anomalies (Figure 3A). Inter-rater reliability was assessed using the intraclass correlation coefficient (ICC) for radiographic grading (ICC = 0.87, 95% CI: 0.79–0.93), indicating good to excellent agreement. The quantitative results (Figure 3B) revealed that the MSU/LPS + Saline group exhibited grade 3 lesions: characterized by extensive soft tissue swelling, periarticular erosion, and calcification formation (marked by red arrows), which was in accordance with ongoing inflammation and crystal deposition. The MSU/LPS + cHA gel group changes pattern was between grade 1 and grade 2, with partial reduction in soft tissue thickening and less calcification (marked by red arrows), indicating moderate protective effects on the affected tissues. Compared with the MSU/LPS + cHA gel group, the MSU/LPS + cHA-Dex gel group showed grade 0–1 changes, with significant reduction in edema, very few bone erosions, almost intact joint contour, and no obvious calcification.

### 3.4 Effect of cHA-Dex gel on gross morphology of knee joints and histopathological outcomes of cartilage in GA rats

**FIGURE 4:**
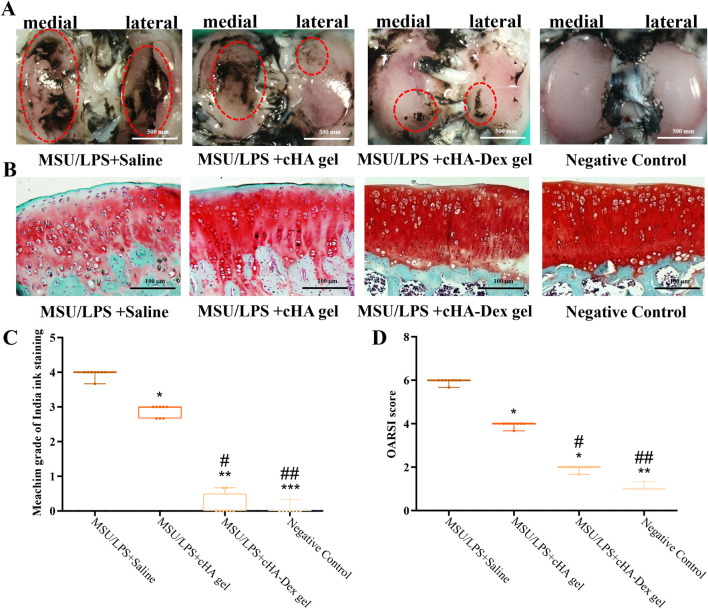
**(A)** Rat tibial plateaus were collected from various experimental groups. Gross morphological damage and fibrosis of the articular cartilage were observed by India ink staining. **(B)** Articular cartilage samples from the tibial plateaus of different experimental groups were sectioned and stained with safranin O–fast green. **(C)** The Meachim scoring system was used to quantitatively assess cartilage damage and fibrosis. **(D)** Cartilage damage and proteoglycan (PG) content were quantitatively assessed using the OARSI histological scoring system. Data are presented as mean ± SD. *p < 0.05, **p < 0.01, and ***p < 0.001 compared to the MSU/LPS + Saline group; ##*p* < 0.01 and ###*p* < 0.001 compared to the MSU/LPS + cHA gel group; n = 8.

To visualize the gross morphological damage and fibrosis of the cartilage in the tibial plateau of rats, Indian ink staining was used (8 rats per group, Figure 4A). The assessments were based on the Meachim grading scale for Indian ink staining (Figure 4C). To assess inter-rater reliability for Meachim grading, the ICC was calculated. The observed ICC of 0.88 (95% CI: 0.81–0.95) reflected good to excellent agreement between different groups. In comparison to the MSU/LPS + Saline and MSU/LPS + cHA gel groups, the MSU/LPS + cHA-Dex gel group showed a lower degree of cartilage damage and fibrosis; however, these indices were significantly higher (*p* < 0.05) in the MSU/LPS + cHA-Dex gel group compared to the negative control group.

Histological changes were evaluated on 1 day after the fifth MSU/LPS induction. Knee joint histopathology showed that both cHA gel and cHA-Dex gel could reduce cartilage injury and degenerative changes. Compared with the MSU/LPS + Saline and MSU/LPS + cHA gel groups, the MSU/LPS + cHA-Dex gel group had a smoother articular cartilage surface, a higher intensity of Safranin O staining, more chondrocytes, less superficial cartilage loss, and a lower fibrosis level. However, these parameters were still worse than those in the negative control group (Figure 4B). Strong fast green staining was found on the joint surfaces of the MSU/LPS + Saline and MSU/LPS + cHA gel groups, indicating extensive joint surface fibrosis; these results were consistent with Indian ink staining. The OARSI cartilage histological grading scores (mean ± standard deviation; Figure 4D) revealed that cartilage deterioration was most extensive in the MSU/LPS + Saline group, whereas the negative control group exhibited the least severe damage (1.10 ± 0.10, *p* < 0.01). The cHA gel and cHA-Dex gel groups showed only slight cartilage deterioration, with corresponding values of 3.80 ± 0.90 and 2.10 ± 0.58 (*p* < 0.01). Inter-rater reliability was assessed using the ICC for OARSI cartilage histological grading (ICC = 0.91, 95% CI: 0.85–0.99), demonstrating good to excellent agreement.

### 3.5 Effect of cHA-Dex gel on expression of COL II, MMP-3, and MMP-9 in the cartilage of GA rats

**FIGURE 5:**
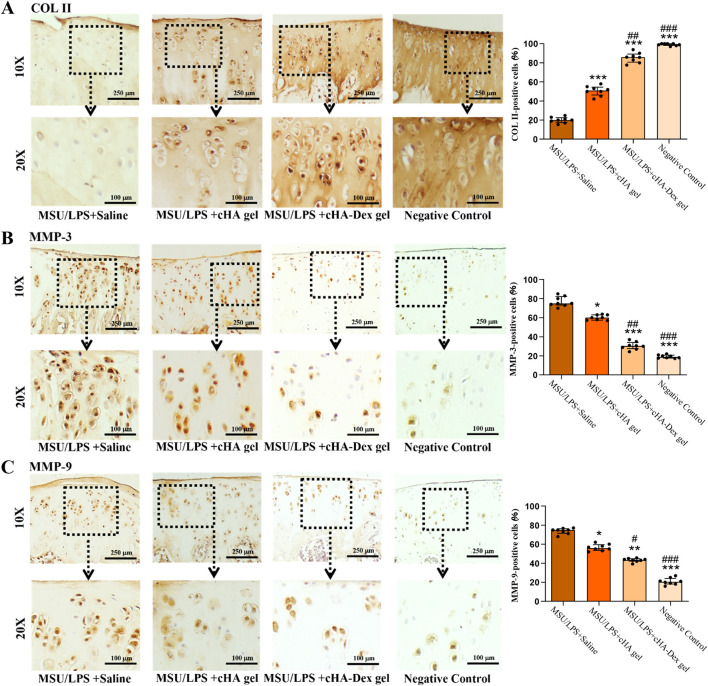
Cartilage sections from the tibial plateau were subjected to immunohistochemical staining to assess the expression levels of type II collagen (COL II) **(A)**, matrix metalloproteinase-3 (MMP-3) **(B)**, and matrix metalloproteinase-9 (MMP-9) **(C)**, positive signals are indicated by brown staining. The expression of each marker was quantified based on the percentage of cells positive for COL II, MMP-3, and MMP-9. Images were acquired at ×10 and ×20 objective magnifications. Data are presented as mean ± SD. *p < 0.05, **p < 0.01, and ***p < 0.001 compared to the MSU/LPS + Saline group; #*p* < 0.05, ##*p* < 0.01 and ###*p* < 0.001 compared to the MSU/LPS + cHA gel group; n = 8.

Immunohistochemical staining was conducted to measure the levels of COL II, MMP-3, and MMP-9 in the cartilage. The MSU/LPS + cHA-Dex gel group demonstrated significantly higher positive COL II staining in cartilage than the MSU/LPS + Saline and MSU/LPS + cHA gel groups (Figure 5A). Regarding the expression levels of MMP-3 and MMP-9 in cartilage, the MSU/LPS + cHA-Dex gel group showed only faint staining, whereas the levels of these two matrix metalloproteinases were moderately elevated in the MSU/LPS + cHA gel group. The MSU/LPS + Saline group showed the highest intensities of MMP-3 and MMP-9 expression (Figures 5B,C). Statistical analysis confirmed that the rates of positive cells for these markers varied significantly among the different treatment groups (*p* < 0.05).

### 3.6 Effect of cHA-Dex gel on synovial inflammation in the knee of GA rats

**FIGURE 6:**
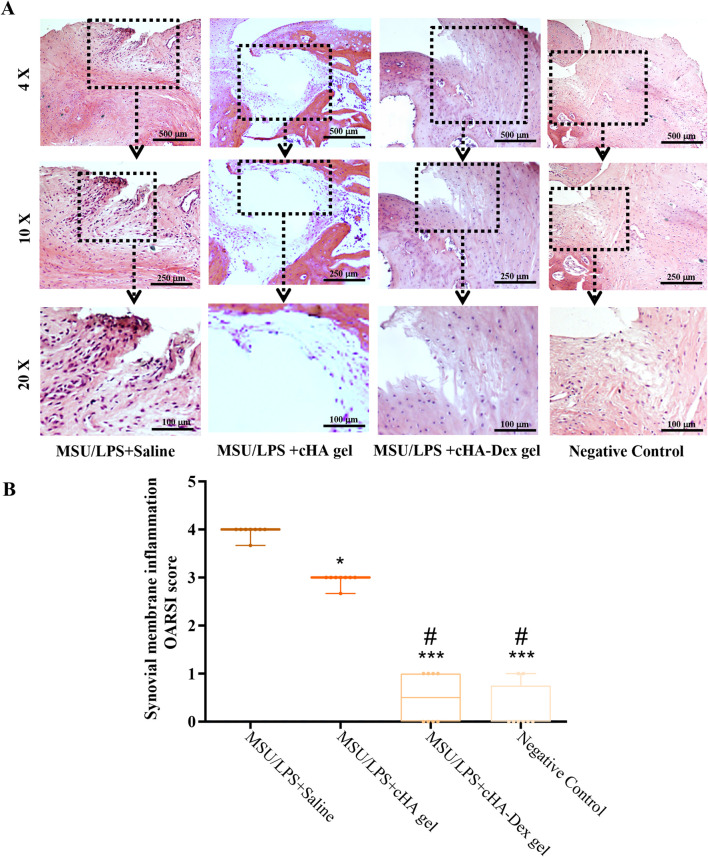
**(A)** Synovial tissues were harvested from rat knee joints and processed for histological sectioning. Inflammatory cell infiltration in the synovial tissue was assessed visually by hematoxylin and eosin (H&E) staining. **(B)** The degree of synovial inflammation was quantitatively assessed using the OARSI synovitis scoring system. Histological images were acquired using objective lenses at magnifications of ×4, 10×, and 20×. Data are presented as mean ± SD. **p < 0.01 and ***p < 0.001 compared to the MSU/LPS + Saline group; ##*p* < 0.01 compared to the MSU/LPS + cHA gel group; n = 8.

H&E staining was conducted on rat knee joint specimens to evaluate the therapeutic efficacy of cHA gel and cHA-Dex gel in alleviating MSU/LPS-induced gouty synovitis. Special attention was given to synovial hyperplasia and inflammatory cell infiltration. Staining results in Figure 6A showed no inflammatory cell infiltration in the negative control group, whereas the MSU/LPS model group exhibited typical synovitis with manifestations including synovial thickening and dense, disorganized inflammatory cell accumulation. Treatment with either gel had a significant effect on the decrease of inflammatory cell infiltration. The synovial tissue of the MSU/LPS + cHA-Dex gel group was nearly normal, with only a few slightly disorganized inflammatory cells, showing greater improvement than the MSU/LPS + cHA gel group. Figure 6B demonstrated that the MSU/LPS model group scored the highest on the OARSI synovitis scale. The inter-rater reliability for OARSI synovitis histological grading was evaluated using the ICC. An ICC value of 0.90 (95% CI: 0.83–0.96) was obtained, indicating good to excellent agreement among those groups. Both cHA gel and cHA-Dex gel treatments led to a significant decrease in this score, with the lowest score observed in the MSU/LPS + cHA-Dex gel group; these differences reached statistical significance (*p* < 0.01). Overall, cHA-Dex gel was more effective in reversing MSU/LPS-induced synovial pathological lesions.

### 3.7 Effects of cHA-Dex gel on the expression of inflammatory mediators in GA rats

**FIGURE 7:**
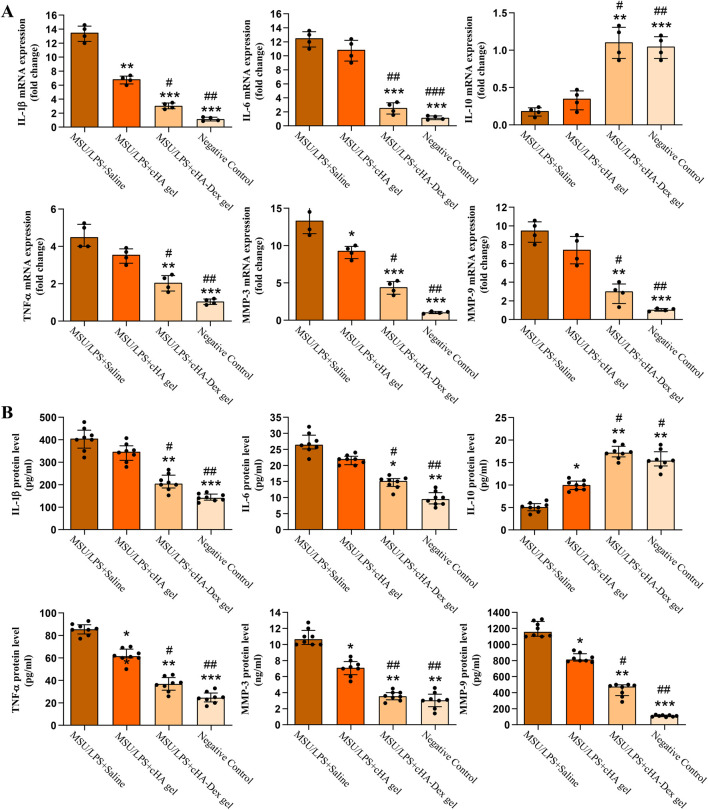
**(A)** Articular cartilage samples were collected for total RNA extraction, and quantitative real-time polymerase chain reaction (RT-qPCR) was used to measure the relative mRNA expression of interleukin-1β (IL-1β), interleukin-6 (IL-6), interleukin-10 (IL-10), tumor necrosis factor-α (TNF-α), matrix metalloproteinase-3 (MMP-3), and matrix metalloproteinase-9 (MMP-9); n = 4. **(B)** One day after the fifth MSU/LPS induction, rats were euthanized and abdominal aortic blood was collected. After centrifugation, enzyme-linked immunosorbent assay (ELISA) was used to quantify serum protein levels of cytokines and matrix metalloproteinases; n = 8. Data are presented as mean ± SD. *p < 0.05, **p < 0.01, and ***p < 0.001 compared to the MSU/LPS + Saline group; #*p* < 0.05, ##*p* < 0.01, and ###*p* < 0.001 compared to the MSU/LPS + cHA gel group.

Serum samples were collected from GA rats injected with MSU/LPS and treated with cHA gel or cHA-Dex gel, and the protein levels of IL-1β, IL-6, TNF-α, MMP-3, and MMP-9 were measured (Figure 7A). Rats with MSU/LPS-induced arthritis displayed markedly higher levels of IL-1β, IL-6, TNF-α, MMP-3, and MMP-9 compared to the negative control group (*p* < 0.05), with respective upregulations of 1215%, 1103%, 321%, 1251%, and 822%. A notable 84.33% decrease in IL-10 protein level was observed in these arthritic rats. Both cHA gel and cHA-Dex gel treatment significantly downregulated the expression of IL-1β, IL-6, TNF-α, MMP-3, and MMP-9, along with a dramatic increase in IL-10 expression, with cHA-Dex gel showing a superior and therapeutically evident effect. These data indicated that cHA-Dex gel has greater anti-inflammatory potential in downregulating MSU/LPS-induced inflammation in GA rats. The analysis of cytokine mRNA expression (Figure 7B) was in accordance with the ELISA data described above.

## 4 Discussion

Gout is a disease that results from persistent purine metabolic disorders and high serum uric acid levels, which eventually cause tissue damage. The main clinical features of gout are hyperuricemia, repeated episodes of acute arthritis, tophus formation, and the development of chronic arthritis with joint deformities (Liu et al., 2025). Globally, about 1% of adults suffer from gout, and GA is the most common type of arthritis in males (Webster, 2024). In the development of GA, a large number of inflammatory factors, including ILs, MMPs, and TNF-α, are released into the joint space (Ouyang et al., 2021). These factors lead to chondrocyte apoptosis and disrupt the proteoglycan (PG) and collagen networks of articular cartilage (Wilson and Saseen, 2016; Keller and Mandell, 2022). Repair and regeneration become almost impossible after the extracellular matrix of articular cartilage is damaged by inflammation (Xu et al., 2023). Therefore, preventing the chronic inflammatory reaction in GA could be a strategy to reverse early cartilage damage, thereby delaying disease progression (Liu et al., 2023).

Behavioral experimental data showed that cHA-Dex gel greatly decreased swelling of the knee joint in GA rats. In addition, cHA-Dex gel-treated rats showed improvement in hindlimb weight-bearing imbalance, grip strength, mechanical pain threshold, and cold hyperalgesia (*p* < 0.05). By modulating these parameters, cHA-Dex gel application recovered locomotor activity defects in GA rats. These results indicated that cHA-Dex gel can effectively counteract behavioral changes in GA rats.

GA is strongly linked to hyperuricemia. It is characterized by the deposition and formation of MSU crystals in the joints and other periarticular tissues (Liu et al., 2025). Epidemiological studies have shown that chondrocalcinosis can occur in gout patients, with the knee joint being the most frequently affected site (Ciancio et al., 2012). Cartilage calcification can appear as linear or irregular distributions (Ciancio et al., 2012). In addition to the knee joint, calcification can also occur in the symphysis pubis, hip joint, and wrist joint (Yin et al., 2014). This phenomenon may result from a common predisposition to connective tissue crystal deposition or be closely related to cartilage damage (Stockman et al., 1980; Ciancio et al., 2012). Micro X-ray imaging revealed that intra-articular administration of cHA gel or cHA-Dex gel significantly reduced inflammatory knee joint swelling, lowered the risk of joint margin erosion, and decreased the number of calcified foci compared with the normal saline group. The cHA-Dex gel group showed more pronounced ameliorative effects. Multiple detection methods, including India ink staining, safranin O–fast green double staining, ELISA, and RT-qPCR, consistently demonstrated that cHA-Dex gel treatment downregulated multiple inflammatory factors, retarded cartilage fibrosis development, and significantly inhibited the degradation of COL II and PG. Most importantly, cHA-Dex gel was significantly more effective in reducing synovial inflammation than either cHA gel or normal saline. Together, the imaging and biochemical data showed that cHA-Dex gel exerted anti-inflammatory and chondroprotective effects in the GA model, with therapeutic efficacy superior to that of cHA gel alone.

One of the major roles of HA, present in synovial fluid and cartilage matrix, is to maintain joint physiological homeostasis by regulating its concentration (Marinho et al., 2021). When exogenous HA is introduced into the joint cavity, it forms a lubricating and cushioning membrane on the cartilage surface, leading to improved nutrient diffusion, decreased mechanical friction, and ultimately slowed cartilage tissue degradation (Familiari et al., 2023). Furthermore, several studies have confirmed that HA can mask exposed nerve terminals, thereby reducing pain hypersensitivity (Ferkel et al., 2023). Currently, intra-articular administration of HA is a standard clinical practice for relieving knee pain (Ran et al., 2018). According to the evidence from the present investigation, cHA-Dex gel was able to inhibit the secretion of IL-1β, IL-6, TNF-α, MMP-3, and MMP-9 to a great extent (Petterson and Plancher, 2019; Smith et al., 2019). These inflammatory mediators can degrade PG in articular cartilage. Among them, MMP-3 and MMP-9 are considered the main contributors to articular cartilage destruction (Hsieh et al., 2003; Shi et al., 2021). MMP-3 and MMP-9 expression levels were higher in the group treated with cHA gel alone compared to the group treated with cHA-Dex gel. The results of this experiment suggest that cHA gel is not highly effective in exerting significant anti-inflammatory activity, whereas cHA-Dex gel can potently suppress MMP-3 and MMP-9 secretion, thereby facilitating the recovery of COL II and PG expression in GA. While the present study demonstrated reduced MMP-3 and MMP-9 expression at the mRNA and protein levels, we did not assess enzymatic activity directly. Future work incorporating zymography or activity-based assays would help determine whether the observed reductions translate into a decreased functional degradative capacity. The observed anti-inflammatory and chondroprotective effects of cHA-Dex gel are likely mediated by modulation of GA-relevant inflammatory and catabolic pathways, which require further mechanistic characterization. The molecular analysis focused on pro-inflammatory cytokines, IL-10, and MMP-3/9 as key endpoints. However, additional pathways such as NLRP3 inflammasome activation (NLRP3, ASC, and caspase-1), NF-κB signaling, and aggrecanases (ADAMTS-4/5) were not assessed. ADAMTS-4 and ADAMTS-5, the principal aggrecanases in cartilage degradation, should also be assessed. Future mechanistic studies will address these pathways to more comprehensively define the mode of action of cHA-Dex gel in GA.

Long-term, repeated administration of high-dose Dex is well known to markedly inhibit chondrocyte proliferation and accelerate their apoptosis. In contrast, a single intra-articular administration of a very low concentration of Dex has been shown to delay disease progression in GA by suppressing early inflammatory responses (dos Santos et al., 2023). However, these formulations are typically short-acting and may require repeated injections, especially in the management of recurrent GA (dos Santos et al., 2023). Experimentally, Dex has been shown to suppress the progression of inflammatory joint diseases through anti-inflammatory mechanisms. The combined use of Dex and cHA can result in high therapeutic efficacy (Zhang et al., 2016). Synvisc, a macromolecular HA gel widely used in clinical practice, can effectively alleviate pain symptoms of GA after intra-articular injection (Marcotti et al., 2018). In the present study, we developed a crosslinked, premixed cHA-Dex polymer gel that is resistant to degradation by multiple enzymes and exhibits prolonged biological activity, which was administered via a single intra-articular injection for GA treatment. The benefit of cHA was enhanced when used in combination with Dex, which inhibits inflammatory reactions and decreases cytokine production. A Dex-loaded cHA gel can gradually release Dex into the joint cavity, thereby exerting therapeutic effects. Although intra-articular injection was performed unilaterally, the potential for systemic absorption of dexamethasone or hyaluronic acid affecting the contralateral limb cannot be entirely excluded. However, given the low dose and localized administration, such effects are likely minimal and would not substantially alter the asymmetry-based behavioral assessments. Combination products incorporating hyaluronic acid and corticosteroids have been explored in other arthritic conditions, though their application in GA remains limited (Gupta et al., 2025). The present study contributes to this landscape by evaluating a cHA-Dex formulation in a repeated GA model, although further comparative studies with existing clinical preparations are needed to establish relative efficacy.

This study has several limitations that are worth pointing out. A notable limitation of this study is the absence of a control group receiving Dex alone (0.5 mg/mL). Without this comparator, the relative contributions of the pharmacologic action of Dex, the potential synergistic interaction with the cHA matrix, and the sustained-release properties of the hydrogel cannot be fully delineated. The decision to omit this group was based on prior evidence from our 2016 investigation (Zhang et al., 2016) as well as the 2024 mechanistic follow-up (Zhang et al., 2024), wherein cHA-Dex demonstrated superior chondroprotection compared with both cHA alone and Dex alone in chondrocytes. Our 2016 investigation (Zhang et al., 2016) has confirmed that chondrocyte viability markedly decreases when the Dex concentration reaches 13.3 μg/mL. A systematic review by Black et al. pointed out that Dex has a “double-edged sword” effect on chondrocytes—low doses offer protection while high doses cause damage (Black and Grodzinsky, 2019). However, the regulatory strategies for its safety window remain unclear.

Our 2024 investigation (Zhang et al., 2024) has demonstrated the potential delivery advantage of cHA-Dex gel relative to current treatments. During the first few days of the experiment, the amount of Dex released from the cHA-Dex gel was relatively high. Nevertheless, when compared with the Dex-only group, no pronounced burst release of Dex was observed in cHA-Dex gel. In the following days, Dex release became remarkably stable and remained at a low sustained level over a long period in cHA-Dex gel. This behavior is thought to arise from the pre-mixed cHA-Dex gel’s intrinsic structure—a three-dimensional network formed through polymer self-crosslinking. Such a network appears to delay the concentrated, abrupt release of Dex, promoting instead a slow and low-dose release pattern. As a result, the adverse effects typically associated with Dex, including increased chondrocyte apoptosis and reduced proliferation rates, are substantially minimized or even eliminated. Meanwhile, the sustained anti-inflammatory action of Dex can be more realized. Consistent with this interpretation, the experimental data showed that, relative to the Dex-only group, treatment with the cHA-Dex gel led to decreased chondrocyte apoptosis (0.60 ± 0.07 vs. 6.63 ± 0.98) and enhanced chondrocyte proliferation (P < 0.05 for each timepoint). These findings supported the conclusion that the cHA-Dex gel achieved a sustained release of Dex, and also reflected the beneficial outcome of Dex’s long-term anti-inflammatory activity. However, the sustained-release narrative rests upon prior pharmacokinetic characterization of cHA-Dex gel (Zhang et al., 2024) rather than direct measurement in this study. We have initiated high-performance liquid chromatography analyses of synovial fluid Dex concentrations in a parallel GA study, measuring Dex levels in synovial fluid and joint tissue at 1, 7, 14, 28, and 56 days post-injection. Our ongoing studies will include a dose-response analysis with different Dex concentrations (0.2–1.0 mg/mL) to define the therapeutic window for cHA-Dex gel in GA, including assessments of anti-inflammatory efficacy, chondroprotection, and potential Dex-induced chondrocyte toxicity at each dose.

Another limitation of this study is that all outcome measures were evaluated at a single endpoint. Consequently, the durability of the therapeutic response and the temporal relationship between drug release and disease progression could not be fully characterized. The current study is our first step to investigate the general efficacy of cHA-Dex gel in the treatment of repeated GA. Another ongoing study incorporating intermediate assessments after the second, third, or fourth induction cycles would help clarify the kinetic profile of the sustained-release formulation.

The third limitation is regarding the pooling of cartilage samples for RT-qPCR: this approach was adopted due to the limited amount of cartilage tissue obtainable from the rat femoral condyle. The pooling strategy (two rats per pool) was implemented to ensure sufficient RNA yield for reliable quantification. We have ensured that statistical analyses for molecular data are based on the number of pooled samples (n = 4 per group), with technical replicates (six repeats) used to confirm reproducibility. Our follow-up studies will use larger cartilage tissue samples (e.g., from rabbit GA models) to avoid pooling and preserve individual variability.

This study utilized only male rat model for testing, the lack of bipedal locomotion may limit the applicability of the findings to humans. Although GA is more prevalent in males, the condition also significantly affects females, particularly after menopause, and sex hormones may modulate inflammatory responses and cartilage metabolism. This study utilized young adult (2-month-old) rats, whereas GA predominantly affects older individuals. Aging is associated with altered cartilage regenerative capacity, increased baseline inflammation, and changes in drug metabolism, all of which may influence treatment responses. The present findings should therefore be considered proof-of-concept in a young-animal model. Moreover, the LPS-amplified inflammatory baseline may overestimate anti-inflammatory efficacy and the model does not fully recapitulate human GA pathophysiology. To overcome these limitations, an aged minipig model of GA, including both sexes, has been employed to confirm the therapeutic effects of cHA-Dex gel in a more physiologically relevant setting in another preclinical study. Besides, the pathological features of GA involve multiple joint tissues: in addition to articular cartilage lesion and synovial inflammation, subchondral bone, the joint capsule, and other components also play significant roles in disease progression. The present study focuses mainly on the anti-inflammatory and chondroprotective effects of cHA-Dex gel and does not address urate crystal deposition or urate-lowering mechanisms—core components of clinical GA management. In the ongoing minipig model, we aim to further investigate how cHA-Dex gel affects these additional joint tissues by assessing joint urate crystal burden via micro-CT and synovial fluid urate concentration measurements to evaluate potential indirect effects on crystal clearance. The application of cHA-Dex gel in repeated GA is still at an early research stage, with only a limited number of relevant academic publications available. This paper is only a preliminary step towards the treatment of repeated GA with cHA-Dex gel, and the molecular basis of its therapeutic action needs to be profoundly studied.

## 5 Conclusion

**FIGURE 8:**
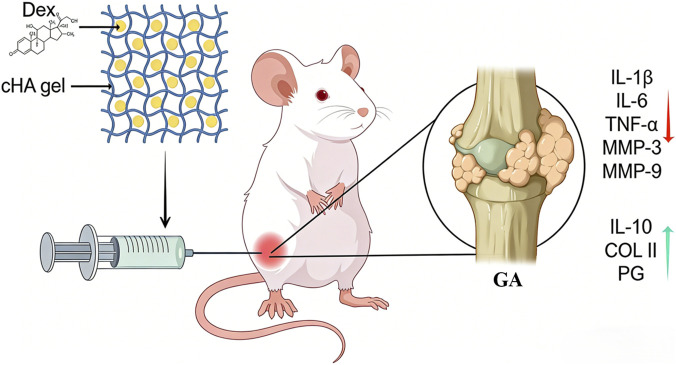
As shown in the diagram, cHA-Dex gel significantly decreased the expression levels of interleukin-1β (IL-1β), interleukin-6 (IL-6), tumor necrosis factor-α (TNF-α), matrix metalloproteinase-3 (MMP-3), and matrix metalloproteinase-9 (MMP-9) in the knee joints of rats with gouty arthritis (GA). Simultaneously, it increased the protein level of interleukin-10 (IL-10). These changes ultimately resulted in reduced cleavage of type II collagen (COL II) and proteoglycan (PG) in cartilage of GA rat (This figure was drawn by Figdraw).

In summary, the current study verified that cHA-Dex gel could alleviate recurrent MSU/LPS-induced GA by suppressing the expression of IL-1β, IL-6, TNF-α, MMP-3, and MMP-9, while markedly increasing the expression of IL-10, COL II, and PG (Figure 8). Meanwhile, our findings also revealed that cHA-Dex gel exerted more favorable therapeutic effects than cHA gel alone in a rat model of repeated GA, providing an adjuvant strategy that may complement current clinical approaches for recurrent GA.

## Data Availability

The original contributions presented in the study are included in the article/Supplementary Material, further inquiries can be directed to the corresponding authors.
