# Supplementary material for: Anti-arthritic activities of cross-linked hyaluronic acid-dexamethasone hydrogel in a rat model of gouty arthritis
Source: Front Pharmacol. 2026 May 13;17:1810048. doi: 10.3389/fphar.2026.1810048 (PMC13212335; doi:10.3389/fphar.2026.1810048)
Supplement: Supplementary file 1 [file Table1.docx]

Supplementary Material

**Supplementary Table S1** Primer sequences used for RT-qPCR in this study.

| **Genes** | **Forward primer** | **Reverse primer** |
| --- | --- | --- |
| 18S | CGGCTACCACATCCAAGGAA | GCTGGAATTACCGCGGCT |
| IL-1β | AGGAGAGACAAGCAACGACA | CTTTTCCATCTTCTTCTTTGGGTAT |
| IL-6 | AGCGATGATGCACTGTCAGA | TAGCACACTAGGTTTGCCGA |
| IL‐10 | AGGGTTACTTGGGTTGCC | GGGTCTTCAGCTTCTCTCC |
| TNF-α | ACTGAACTTCGGGGTGATCG | GCTTGGTTTGCTACGAC |
| MMP-3 | TGGGCTATCCGAGGTCATG | TGCCTGGAAAGTTCTCAGCTATT |
| MMP-9 | AGGATGGTCTACTGGCACAC | GTGCAGGACAAATAGGAGCG |

IL-1β: interleukin 1 beta; IL-6: interleukin 6; IL-10: interleukin 10; TNF-α: tumor necrosis factor-alpha; MMP-3: Matrix metalloprotease 3; MMP-9: Matrix metalloprotease 9.
